# Comparative molecular dynamics simulations of thermal conductivities of aqueous and hydrocarbon nanofluids

**DOI:** 10.3762/bjnano.13.54

**Published:** 2022-07-07

**Authors:** Adil Loya, Antash Najib, Fahad Aziz, Asif Khan, Guogang Ren, Kun Luo

**Affiliations:** 1 National University of Sciences and Technology, Department of Mechanical Engineering, H-12, Islamabad, Pakistan; 2 Karachi Institute of Economics and Technology, Department of Mechatronics Engineering, Karachi, Pakistan; 3 Aeroprecinyx, Karachi, Pakistan; 4 University of Hertfordshire, School of Engineering and Technology, Hatfield, UK; 5 Changzhou University, School of Materials Science and Engineering, Changzhou Science Town, Changzhou, P. R. China

**Keywords:** alkanes, aqueous solutions, CuO, hydrocarbon solutions, molecular dynamics simulation, nanoparticles, thermal conductivity

## Abstract

The addition of metal oxide nanoparticles to fluids has been used as a means of enhancing the thermal conductive properties of base fluids. This method formulates a heterogeneous fluid conferred by nanoparticles and can be used for high-end fluid heat-transfer applications, such as phase-change materials and fluids for internal combustion engines. These nanoparticles can enhance the properties of both polar and nonpolar fluids. In the current paper, dispersions of nanoparticles were carried out in hydrocarbon and aqueous-based fluids using molecular dynamic simulations (MDS). The MDS results have been validated using the autocorrelation function and previous experimental data. Highly concurrent trends were achieved for the obtained results. According to the obtained results of MDS, adding CuO nanoparticles increased the thermal conductivity of water by 25% (from 0.6 to 0.75 W·m^−1^·K^−1^). However, by adding these nanoparticles to hydrocarbon-based fluids (i.e., alkane) the thermal conductivity was increased three times (from 0.1 to 0.4 W·m^−1^·K^−1^). This approach to determine the thermal conductivity of metal oxide nanoparticles in aqueous and nonaqueous fluids using visual molecular dynamics and interactive autocorrelations demonstrate a great tool to quantify thermophysical properties of nanofluids using a simulation environment. Moreover, this comparison introduces data on aqueous and nonaqueous suspensions in one study.

## Introduction

The term nanofluids denotes solid nanoparticles (1 to 100 nanometres in size) homogenously suspended in a fluid to form a conjugate suspension liquid [1–2]. The use of nanoparticles in a fluidic suspension is not a new practise and can be traced back to over two decades ago [3]. Since the publication by Choi et al. [3] in 1995, nanofluids have been extensively studied since the addition of nanoparticles significantly enhances the heat-transfer performance of the base fluid [4–6]. This has promoted various applications of nanofluids in a wide range of fields, such as cooling fluids for nuclear reactors [7] and for thermal management of electronics [8–9]. As mentioned above, nanofluids have also proved to be very effective as working ﬂuids [10–11] in solar thermal systems and for enhancing the thermal characteristics of phase-change materials (PCM) that are used for latent thermal storage media [12] in solar thermal systems.

In all these applications, nanoparticles act as a means for enhancing the overall thermal conductivity of the base fluid, which consequently improves heat conduction through the system. It is therefore vital to quantify this enhancement in the thermal conductivity, which is only possible if the understanding of the underlying mechanisms of heat transfer in nanofluids is clearly underpinned. Several mechanisms have been suggested by researchers to effectively predict this improvement in thermal conductivity. The most widely accepted mechanisms for dispersion are: a) Brownian motion, b) liquid–liquid layering, c) particle–liquid layering, and d) thermal transfer [1,13].

Initially, it was suggested by several studies that the increased conductivity of nanofluids was due to the Brownian motion of nanoparticles in the fluid [14–15]. It was also proposed that a local micro-convection is induced in the base ﬂuid due to the Brownian motion of nanoparticles, which increases both mixing and heat transport within the nanofluid [16–17]. Later, several studies demonstrated that interactions between liquid atoms and nanoparticles (i.e., a liquid adsorption layer at the liquid–particle interface) is another significant mechanism for heat transfer enhancement [18–20]. Furthermore, a number of researchers have shown that agglomeration (or clustering) of nanoparticles is another key factor that affects the thermal conductivity of nanofluids [21–23]. However, no one can visualize these phenomena as they take place at very low levels.

Although many studies have been carried out in the past, determining the complexities of various factors at the nanoscale is challenging (i.e., limitless combinations and configurations of nanoparticles and base fluids) and further research in this area is required. In addition to this, it is known through experiments and observations that the volume fraction of nanoparticles is the most important property that enhances the thermal conductivity of a fluid, and this increment is linear. Secondly, the shapes of nanoparticles change thermal conductivity, as investigated by Zhu et al. [24]. In their research, CuO nanowires and nanospheres were dispersed in a dimethicone base fluid, and it was found that changes in the crystalline structure of nanoparticles change the kinetics of the base fluid [24]. In addition to this, it was further investigated that the thermal conductivity increases more with CuO nanowires than with CuO nanospheres. Only 6.98% of improvement was recorded with nanospheres, while 60.78% was recorded with nanowires. Ibrahim et al. [25] conducted a study using a neural network model (ANN) to study the extended theoretical observation of thermal conductivity and its correlation with different percentages of mass fraction of graphene nanosheets loaded into ethylene glycol (EG). An increasing trend of the thermal conductivity of the nanofluid was observed (i.e., from 0.246 to 0.251 W·m^−1^·K^−1^ at 100% growth). Chu et al. [26] tested the validity of RSM and ANN algorithms for predicting relative thermal conductivity and compared it with experimental data points. Only a 0.3% error was noted, which proves the effectiveness of both models.

It was found that the thermal conductivity of water is increased by inducing CuO nanoparticles. A study was conducted in which it was observed that the CuO/water nanofluid had a positive impact, which resulted in an enhancement of thermal conductivity of about 12.4% as compared to distilled water. The KD2 thermal property analyzer was used for the measurements. Initially, the thermal conductivity of distilled water was found to be 0.611 W·m^−1^·K^−1^ and then, with a CuO nanofluid, 0.698 W·m^−1^·K^−1^ was measured [27]. Another similar experiment was conducted in a different environment. Here, the thermal conductivity and heat-transfer rate of CuO/distilled water nanofluid were analysed. This nanofluid was used in a heat exchanger with small cooling spaces for observing changes in these two thermal properties and it was noted that both thermal properties improved significantly. The concentration of nanofluids used was about 0.27% [28].

Nanofluids can be utilized as a coolant for industrial applications in which particular areas are heat exchangers, (e.g., high horsepower cars, spacecrafts, and satellites). A new geometrical study was carried out in which an I-shaped block was concentrated with CuO/water nanofluid and a triangular hot block with four different orientations was tested [29]. Cooling properties of the CuO/water nanofluid were observed at each orientation. Moreover, efficient and accurate mathematical models were used to obtain readings that were precisely similar to experimental values. A maximum heat transfer rate was recorded in the right-oriented hot block in the sand-based porous cavity with an average percentage of 17.75% [29]. Astanina et al. investigated the utilization of a two-phase nonhomogeneous model (numerical model) of a CuO/water nanofluid for natural convection cases in a partially heated, square-shaped geometry [30].

Heat transportation analysis can be done by the Cattaneo–Christov theory. The inspection of thermal properties of nanofluids can also be carried out via numerical method models built in MATLAB. One of the most prominent investigations of nanofluids using MATLAB was conducted by Abid et al. [31]. They considered Cu/CuO–water and Cu/CuO–kerosene oil ionized nanofluid flow over a stretched three-dimensional linear sheet. The dominant thermal conductivity increment was observed in Cu/water nanofluids in contrast to other partially ionized nanofluids.

For parabolic stretched surfaces [32], a similar theoretical study was carried out with different shapes (i.e., cylinder, platelet, and sphere) of nanoparticles (Cu/Al_2_O_3_ with ethylene glycol as the base fluid) using the finite element method (FEM) in MAPLE 18.0. For mathematical modelling and simulation of hybrid nanofluids, Shah et al. [33] considered a two-dimensional free convective hybrid nanofluid (Fe_3_O_4_ + MWCNT/H_2_O) stream over a resilient cylinder under the influence of a light magnetic field. The heat transportation problem was resolved by combining two methods (FEM and FVM) and an understanding that the temperature near the wall escalated due to an increasing number of nanoparticle collisions was obtained.

It is known from the aforementioned literature that the properties of nanoparticles assist in enhancing the thermal conductive properties of base fluids. In a similar aspect, it was also found that hybridized nanoparticles considerably improve the conductive properties of base fluids. Raja et al. [34] carried out an experiment in which a comparative study of the thermal behaviour of normal and hybrid nanofluids (Al_2_O_3_/H_2_O, CuO/H_2_O and Al_2_O_3_–CuO/H_2_O) was observed. Hybrid nanofluids (Al_2_O_3_–CuO/H_2_O) showed greater enhancement in thermal properties than other conventional nanofluids (CuO/H_2_O, Al_2_O_3_/H_2_O). However, the relationship between the increase in volume fraction and improved thermal properties remained linear. They also observed that experimental values were much higher than the values predicted by models presented in the literature. To further investigate thermal properties of different hybrid nanofluids, Singh et al. [35] used theoretical and experimental results of GO–CuO/DW (graphene oxide and copper oxide nanoparticles dispersed in distilled water) and compared those with mononanofluids (i.e., GO/DW and CuO/DW) at different temperatures. The relative thermal conductivity of GO/DW was found to be the highest among the three nanofluids studied with an enhancement of 51.6%. Meanwhile, the hybrid nanofluid also showed significant improvement of 30%. Thus, it is evident that hybrid nanofluids might not be preferred over mononanofluids.

## Motivation

As phase-change materials, alkane-based nanofluids are being used and it is found that as PCM nanofluids of CuO provide enhanced performance. Therefore, CuO nanoparticles in a nonpolar medium can serve as thermal storage materials [36]. Moreover, heat carrier metal/organic nanofluids of methanol and refrigerants have also shown improved thermal conductivity [37].

The increment in various thermal conductivity applications motivated us to conduct this study. It is known that CuO nanoparticles are not only used for enhancement in polar fluids. Currently, it is extensively being used with nonpolar media as well. Moreover, except for Abid et al. [31], there are not many studies which discuss polar and nonpolar fluid thermal conductivities. However, experimental studies rather than simulations have been carried out. In their study they have not used alkanes; rather, they have used kerosene oil as a nonpolar medium.

Although experimental studies have shown that all the aforementioned mechanisms play a role in the enhancement of the thermal conductivity of a nanofluid, to some degree, these processes necessitate microscopic analysis of the heat transfer within and around nanoparticles and base ﬂuid molecules [1]. The molecular dynamics (MD) simulation method is a tool that has been used to effectively predict nanofluid thermal properties with relative accuracy [38–42]. The method relies on computationally solving basic equations of Newton’s laws of motion for interacting particles at the atomic level. Although a lot of research has been dedicated to different molecular dynamics simulations of phase transitions and thermal properties of nanofluids, unlike previous works, this research aims to examine the thermal behaviour of water/CuO and alkane/CuO nanofluids by comparing the behaviour of both nanofluids. Moreover, this research gives insights regarding the accuracy of thermal conductivities of aqueous and nonaqueous fluids predicted by molecular dynamic simulation. Finally, these MD simulation results were compared with experimental data [43] and previously reported simulation results [44]. Moreover, in earlier research, authors investigated the rheological and diffusion properties of a CuO nanofluid in water-based systems [45]. The present study is a continuation of that research and is focused on predicting the thermal conductivities of CuO nanoparticles in aqueous and nonaqueous solutions using MD simulations. The results were validated using experimental data.

## Methodology

The MDS was performed on two distinct systems containing the same type of nanoparticles (i.e., CuO nanoparticles). These particles were dispersed in aqueous (water) and nonaqueous (alkane, i.e., eicosane C_20_H_42_) media. Before conducting simulations, the nanoparticle was created using the Material Studio software (Accryles Inc., USA is the sole proprietor of the Material Studio software produced for performing material/chemical design and analysis). This particle was then dispersed in water and alkanes.

**Figure 1:**
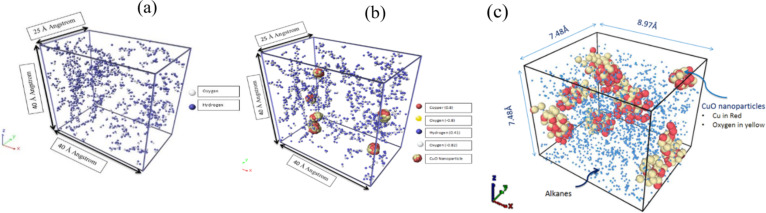
Visualization of various molecular dynamics systems. a) 463 TIP3P pure water molecules in an orthogonal box (i.e., 40 Å × 25 Å × 40 Å), where the white colour represents the oxygen atom and the blue is the hydrogen atom. b) Water/CuO nanofluid in an orthogonal box of 40 Å × 40 Å × 25 Å with 463 water molecules; where red atoms are copper and yellow represents oxygen bonded in a CuO nanoparticle. The blue-coloured atoms are hydrogen and the white-coloured atom is oxygen. c) Alkane/CuO nanofluid, where the cyan blue spheres represent n-eicosane (C_20_H_42_), the red-coloured atoms are Cu atoms, and the yellowish/brown feature is the oxygen connected to Cu atoms.

The large-scale atomic/molecular massively parallel simulator (LAMMPS) molecular dynamic package provided by the Sandia group, created by Plimpton et al. [46–48], was used for simulating the dispersion of nanoparticles in water and alkanes. The water/CuO system consisted of 463 transferable intermolecular potential (3P TIP3P ) water molecules [49]. The simulation was conducted using smoothed particle hydrodynamic (SPH) and discrete particle dynamic (DPD) potentials. The volume of the system was 40 Å × 25 Å × 40 Å, as shown in Figure 1a. Within this system, seven nanoparticles are presented, each constructed with 36 CuO molecules bonded by the COMPASS force field. This constructed nanoparticle size was 0.4 nm, as shown in Figure 1b. A molecular dynamics simulation of paraffin (i.e., eicosane C_20_H_42_) was also conducted for comparison with the aqueous solution. In the alkane/CuO nanofluid simulation, the concentration of CuO nanoparticles was 3% (weight ratio) as shown in Figure 1c.

The desired value calculated for the thermal diffusivity for SPH for CuO NPs in the aqueous fluid was 0.0015719 m^2^/s. The alkane/CuO nanofluid was simulated for approximately 147 picoseconds under NPT (the NPT ensemble enables the system to keep the pressure constant but the volume is varied). The temperature of the nanofluid during simulation was maintained at 303 K with 1 bar pressure. Electrostatic and van der Waals forces were imparted on the nonbonded interaction for dispersion. Charges on the system were incorporated using the COMPASS force field [50]. Moreover, it is known from the literature that the COMPASS force field has already been used for alkanes and benzene-based systems. Therefore, this force field proved to be an accurate approach for the alkane-based fluidic system in this work [50]. The paraffin constructed for molecular dynamics was a straight-chain alkane molecule. The COMPASS force field was applied using the Material Studio. The alkane radius is decreased in the figure for a clearer visualization so that the nanoparticles can be clearly illustrated and identified.

Both systems (i.e., nonaqueous and aqueous-based) were equilibrated from 303 to 323 K, with 10 K steps. All the above simulations were carried out under atmospheric pressure. Dynamical movement of molecules was imparted using smoothed particle hydrodynamic (SPH) and discrete particle dynamics (DPD) potentials. These two potentials (SPH and DPD) were used since they provide a realistic effect on the system configuration. The system was equilibrated for various iteration levels until the heat autocorrelation function demonstrated a monotonic decaying trend.

## Results

**Figure 2:**
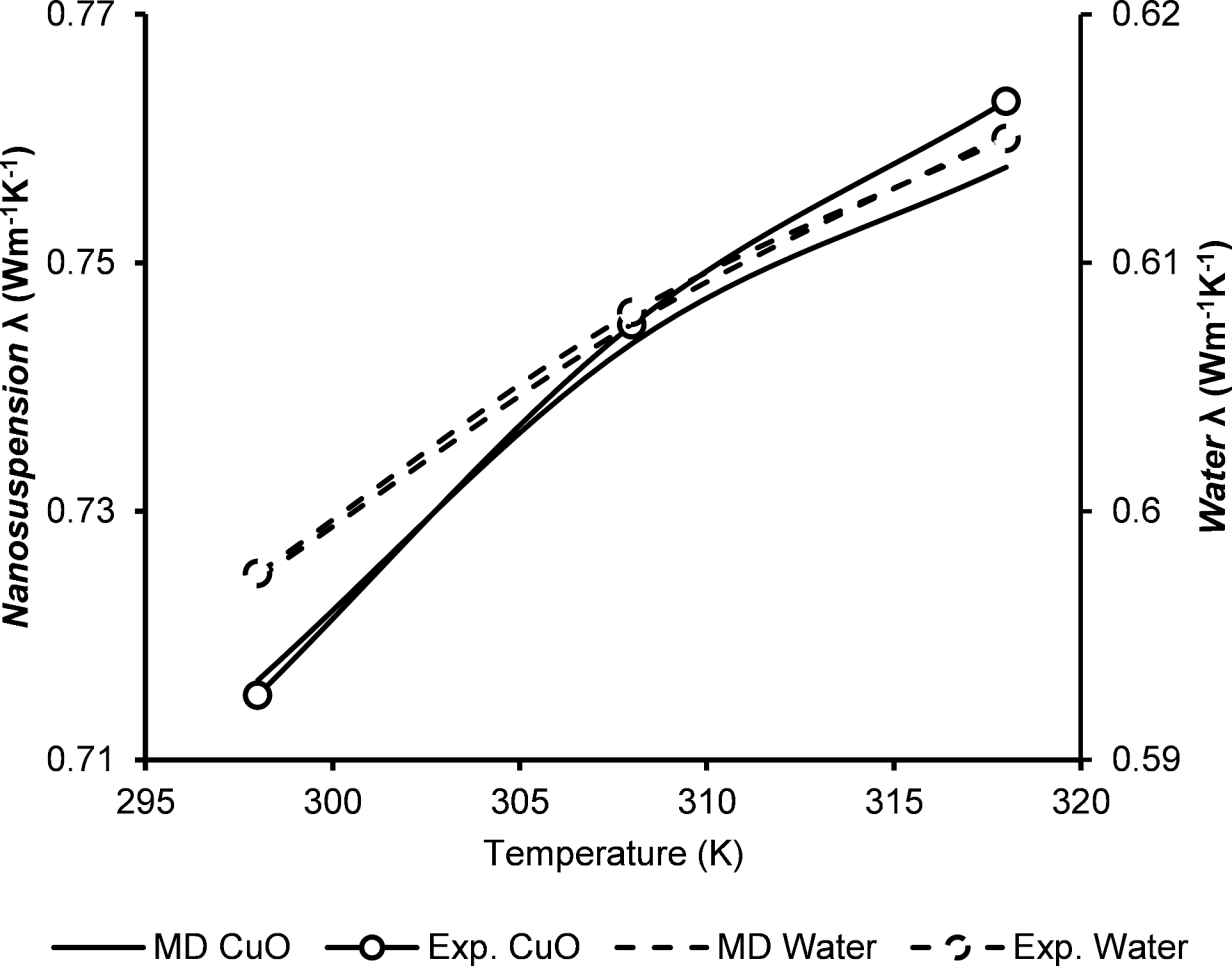
Thermal conductivities of a water-based system and a CuO nanofluid obtained from an experimental study [43] and their molecular dynamics.

In Figure 2, water/CuO nanofluid thermal conductivity results calculated by MDS are presented and compared with experimental results from the research work by Karthik et al. [43]. Karthik et al. used a 3-ω experimental technique to investigate the thermal conductivity of CuO nanofluids. However, in this research, several simulations were conducted at different temperatures with various timesteps to obtain results that are coherent with the experimental findings in Karthik et al. [43]. At 298 K, MDS predicts a thermal conductivity of 0.712 W·m^−1^·K^−1^, which matches well with the results obtained experimentally (i.e., 0.71 W·m^−1^·K^−1^).

Ghasemi et al. [44] used molecular dynamic simulations with EAM and the Dreiding force field to investigate different properties of thermal transport of water/CuO nanofluids. They calculated similar thermal conductivity values for water from 303–323 K, as in our case (i.e., 0.59–0.66 W·m^−1^·K^−1^, respectively). Then they added CuO nanoparticles with various volumetric concentrations in the base fluid (i.e., water) and obtained data with a similar temperature interval as obtained in the current research (i.e., above 0.71–0.92 W·m^−1^·K^−1^) [44]. The MDS results showed that adding CuO nanoparticles enhanced the thermal conductivity of water by 25% (i.e., from 0.6 to 0.75 W·m^−1^·K^−1^).

**Figure 3:**
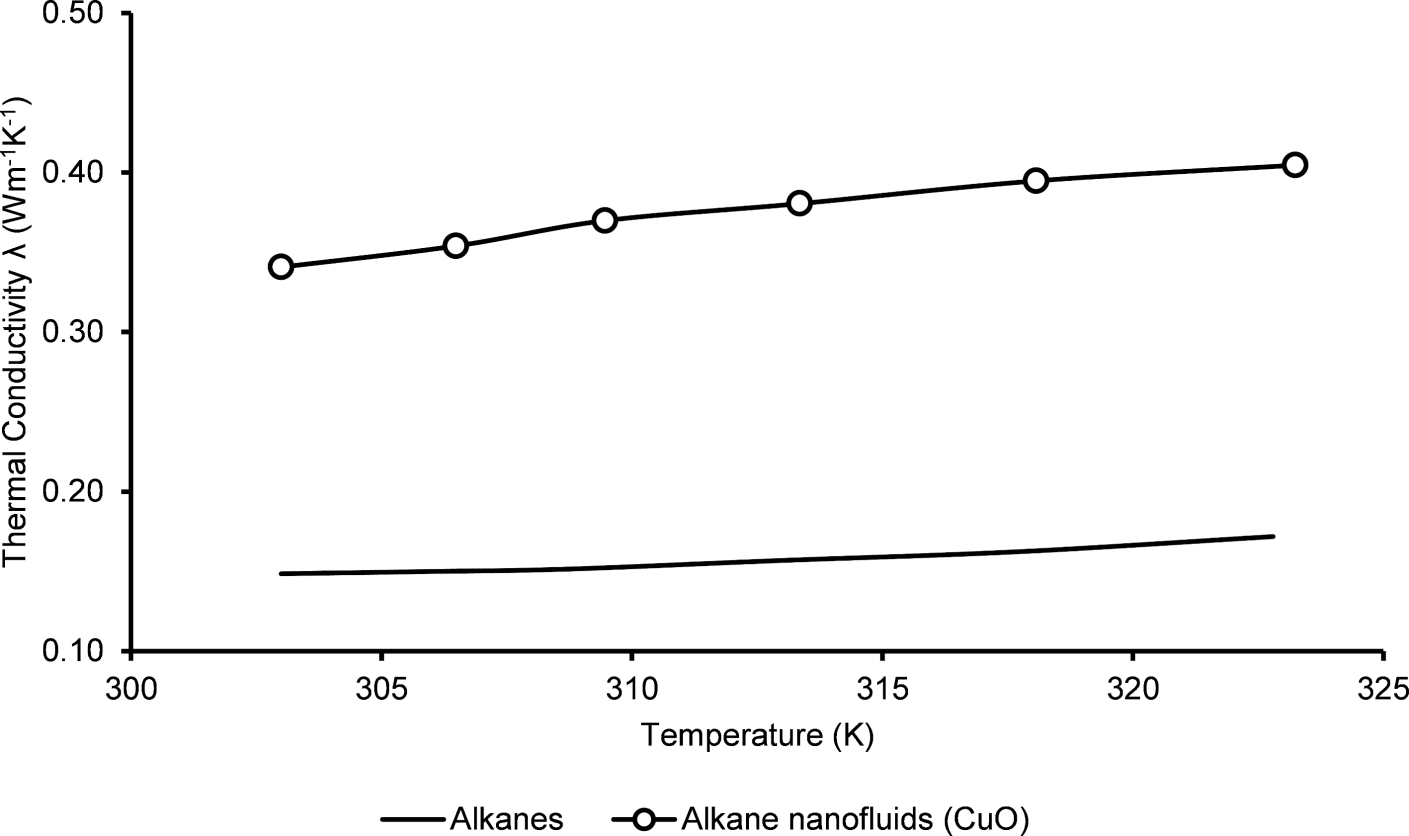
Thermal conductivities of alkanes and alkane/CuO nanofluids obtained from experimental studies and molecular dynamics simulations.

Figure 3 shows the thermal conductivity of pure alkane and alkane/CuO nanofluid systems. The thermal conductivity of pure alkane was used as control. The thermal conductivity in this case was found to be around 0.15 W·m^−1^·K^−1^ at 303 K [51].

**Figure 4:**
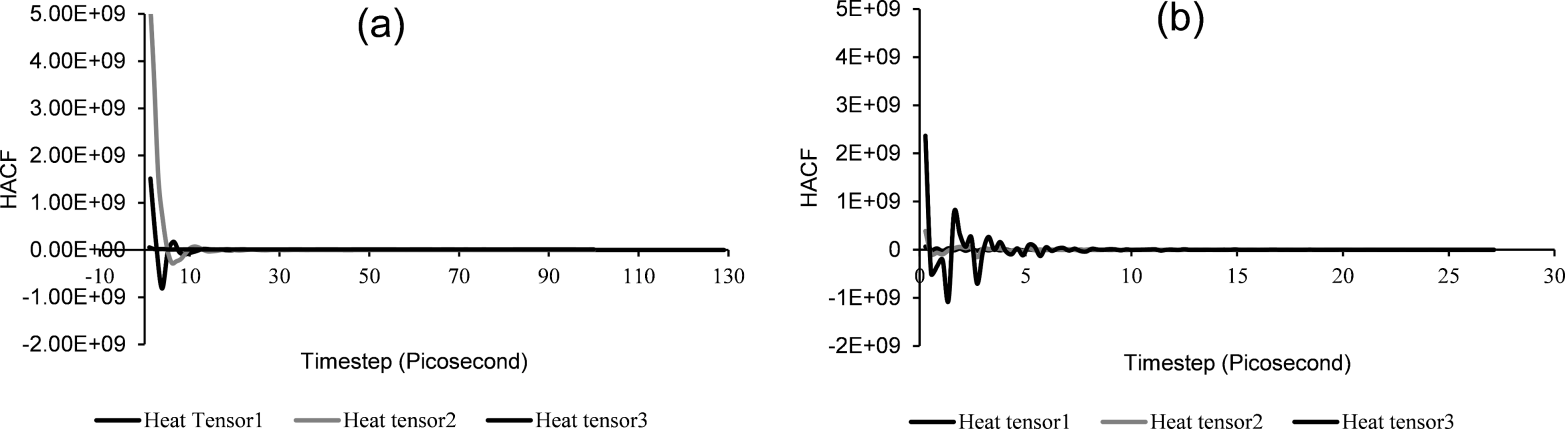
Monotonic decay of the heat autocorrelation functions for a) CuO nanoparticles with water and b) CuO nanoparticle with a hydrocarbon-based fluid.

Later, this system was used for comparison with the nanocluster modified system. As shown in Figure 3, the addition of CuO nanoclusters in the hydrocarbon fluid drastically increases the heat transfer capability. The MDS results demonstrate nearly a 150–200% increase in thermal conductivity properties of the modified system. The high conductivity of water-based nanofluids is due to the higher thermal conductivity of the base fluid itself. However, these results are further explained in the subsequent section with references to heat and current autocorrelation functions (HACF) as represented in Figure 4b.

## Discussions

The methodology adopted for the simulation of CuO/water systems by Ghasemi et al. [44] is different from the methodology used in this study. A comparison is provided in Table 1.

**Table 1:** Comparison of methodologies used for simulating a CuO/water system for thermal conductivity evaluation.

	current study	Ghasemi et al. [44]

formulation	Green–Kubo	Green–Kubo
force field	COMPASS	Dreiding and EAM
potential	SPH and DPD	LJ potential

quantity	thermal conductivity of a nanofluid (W·m^−1^·K^−1^)	thermal conductivity of a nanofluid (W·m^−1^·K^−1^)

	0.720	0.763
	0.746	0.776
	0.760	0.764

It can be seen from Table 1 that the approaches used for simulating nanoparticles with water in this study and in Ghasemi et al. are different. However, the results are in a high degree of agreement with each other. The results of thermal conductivity validation are carried out using heat and current autocorrelation functions. Therefore, for attaining convergence and stability of the readings, it is necessary to achieve the monotonic decay of the function.

Similarly, we obtained the monotonic decay of HACF as shown in Figure 4a and Figure 4b, which demonstrate the stability and reliability of the nanofluid system and of the obtained results. The HACF decay, in general, demonstrates the stability of the system dynamics. The heat and current autocorrelation function is a time-dependent factor which shows that the system stability increases with the simulated time period. At the start of the simulation, HACF shows instability as the system is in an unequilibrated state and the dynamics of the system have not yet started. As the simulation progresses, interatomic collision and dynamics stabilise and the system attains the required temperature profile.

The heat and current autocorrelation function shown in Figure 4a demonstrates that CuO nanoparticles with water stabilised after 30 picoseconds. However, CuO nanoparticles with the hydrocarbon-based fluid took less time to stabilise (i.e., about 10 picoseconds). This is because in the aqueous medium, intermolecular collisions and dynamics of metal oxide atoms form metal ionic bonding, thereby taking more time for equilibration. Moreover, the velocity of the CuO nanoparticles shows high diffusion in water-based systems (i.e., around 4.5 × 10^−9^ m^2^/s) [45], in comparison to alkanes/polar (i.e., nonaqueous, around 4.35 × 10^−11^ m^2^/s) systems [52], as investigated by the two previous studies.

This is further confirmed by a study by Abid et al. [53] in which various parameters (including dimensionless velocity) for water/CuO and kerosene/CuO were calculated using MATLAB. They found that water/CuO nanofluids have a much higher velocity than kerosene/CuO systems. Therefore, both the previous study by the authors regarding diffusion coefficient and the Abid et al. study regarding dimensionless velocity demonstrate that the molecular interaction rate is faster in the water/CuO system as compared to the hydrocarbon-based system.

However, in Abid et al., the thermal conductivity of kerosene/CuO-based nanofluids was found to be higher than that of water-based nanofluids [53]. The key difference was that kerosene was used as a nonaqueous fluid, whereas in the present study paraffin (eicosane, i.e., C_20_H_42_) was used. Nevertheless, in their study, partially ionic systems demonstrated a higher increase in thermal conductance than kerosene-based systems. Therefore, our study demonstrates similar results as Abid et al. (i.e., water/CuO nanofluids have a higher thermal conductivity than alkane/CuO systems).

**Figure 5:**
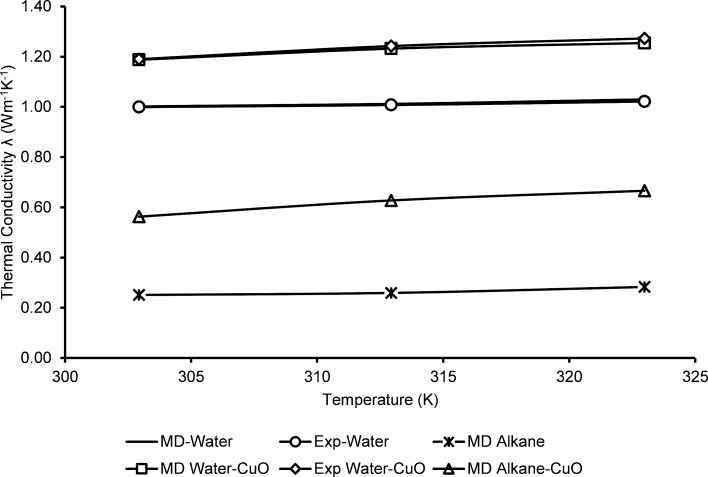
Relative thermal conductivities of various fluids.

Figure 5 shows the relative comparison of the thermal conductivities between hydrocarbon and aqueous systems. Here, water is taken as the reference medium for calculating the relative thermal conductivity of the various fluids. It was found that experimental water/CuO and MD water/CuO trends are in high agreement with each other. However, the thermal conductivity of alkane is lower relative to a water-based system. This is because the thermal properties of the alkanes are very low when compared with pure water. In this study, the thermal conductivity variation for two different nanofluids was conducted without considering the variation in particle concentration. According to a recent study by Topal and Servantie [54], the incremental effect of nanoparticle concentration on the thermal conductivity is valid up to a certain range. However, at a certain point, the incremental effect of the thermal conductivity is halted. Therefore, extensive studies are required in this area to determine the effect of particle concentration on thermal conductivity enhancement. In a similar perspective, optimized alcohol-based nanofluids are being used in internal combustion engines. For this purpose, Cu nanoparticles have been used with ethanol, ethylene glycol, and polypropylene glycol [55].

## Conclusion

This study presents useful information regarding the usage of molecular dynamics simulations for predicting the thermal conductivity of nanofluids for both polar and nonpolar systems. This study showed a high degree of agreement between molecular dynamics and experimental studies [43]. Regarding the heat autocorrelation function and comparative studies, MDS was found to accurately predict thermal properties of the studied systems. The thermal conductivity of water/CuO nanofluids is significantly higher than that of nonpolar nanofluids. It was also found that due to higher velocities, intermolecular collisions and high kinetics of metal oxide atoms happen in aqueous solution, which results in metal ionic bonding. Therefore, the aqueous medium takes longer to equilibrate. The results also show that choosing CuO nanoparticles for modifying aqueous fluids seems to be promising. Analysis of detailed chemical aspects and bonding can further explain the reason why there is an increase in the thermal properties of water/CuO systems. The results of CuO/alkane systems are also useful and have shown an increase in the thermal properties. Moreover, hydrocarbon-based nanofluids are being used in various applications such as PCMs, thermal storage, and coolant for internal combustion engines.

For future considerations, the system under observation should be modified with surfactants to increase stability for longer time periods, as systems without surfactant show agglomeration after a short time. Hence, this causes system destabilisation and creates sedimentation. Finally, surface modification of CuO nanoparticles via molecular dynamics should enable insights on the thermophysical enhancement and stability of CuO nanoparticles in various suspensions.

## Supporting Information

A video of a simulated system of CuO nanoparticles dispersed in water is shown. Frames were processed using the Ovito software. This software is used for simulating molecular dynamics output generated by LAMMPS.

File 1 Video of a simulated system of water with CuO nanoparticles.
